# Pointed Wings, Low Wingloading and Calm Air Reduce Migratory Flight Costs in Songbirds

**DOI:** 10.1371/journal.pone.0002154

**Published:** 2008-05-14

**Authors:** Melissa S. Bowlin, Martin Wikelski

**Affiliations:** Department of Ecology and Evolutionary Biology, Princeton University, Princeton, New Jersey, United States of America; University of Oklahoma, United States of America

## Abstract

Migratory bird, bat and insect species tend to have more pointed wings than non-migrants. Pointed wings and low wingloading, or body mass divided by wing area, are thought to reduce energy consumption during long-distance flight, but these hypotheses have never been directly tested. Furthermore, it is not clear how the atmospheric conditions migrants encounter while aloft affect their energy use; without such information, we cannot accurately predict migratory species' response(s) to climate change. Here, we measured the heart rates of 15 free-flying Swainson's Thrushes (*Catharus ustulatus*) during migratory flight. Heart rate, and therefore rate of energy expenditure, was positively associated with individual variation in wingtip roundedness and wingloading throughout the flights. During the cruise phase of the flights, heart rate was also positively associated with wind speed but not wind direction, and negatively but not significantly associated with large-scale atmospheric stability. High winds and low atmospheric stability are both indicative of the presence of turbulent eddies, suggesting that birds may be using more energy when atmospheric turbulence is high. We therefore suggest that pointed wingtips, low wingloading and avoidance of high winds and turbulence reduce flight costs for small birds during migration, and that climate change may have the strongest effects on migrants' in-flight energy use if it affects the frequency and/or severity of high winds and atmospheric instability.

## Introduction

During a 42 day, 4800 km journey from Panama to Canada, an average Swainson's thrush (*Catharus ustulatus*), a typical passerine migrant, beats its wings approximately 3.2 million times and uses about 1300 kJ of energy on flight alone: energy that would keep it alive almost fifteen days if it did not migrate [1]–[2]. Because flight increases energy expenditure by approximately 50% during the migratory period, natural selection should favor morphology and behavior that decrease these costs to the extent that opposing selective pressures allow [3]–[4]. For example, aerodynamic theory predicts that migrants will benefit from having high aspect ratio wings because they reduce drag during flight [5]–[6]. High aspect ratio is correlated with wingtip pointedness, which is thought to independently increase energy efficiency during flight because individuals with pointed wingtips are believed to shed wake vortices more efficiently than conspecifics [3]. All else being equal, birds with pointed wingtips should thus use less energy than birds with rounded wingtips during sustained, level flapping flight [3]. Similarly, low wingloading, or body mass divided by wing area, is thought by some researchers to decrease the cost of transport for migrants [6]–[7], though this pressure is opposed by the need to carry adequate fuel supplies [8]. Such hypotheses remain untested on free-flying birds [9], but see [10] because previous observational methods revealed nothing about the physiology of individual migrants during natural, uninterrupted migratory flight. What data we do have on the relationship between individual morphology and energy efficiency during flight come from birds flying in wind tunnels [8], [11]–[13] or from examinations of interspecific trends in morphology [3]–[4].

Similarly, it is largely unclear how atmospheric conditions affect the energy expenditure of migrating birds. Aerodynamic theory makes few clear predictions about the effects of atmospheric conditions on energy expenditure during flight except that i) birds should use less energy in tailwinds and more energy in headwinds and that ii) the predicted decrease in power required to fly during the course of long migratory flights due to mass loss will be partially offset by a slight increase in altitude and thus power required to fly. This predicted increase in power is a result of changes in air density and partial pressure of oxygen with increasing altitude [5]–[6], [14]–[16]. Observations by radar, moonwatching, and celiometer beams show increased density of migrants with calm and/or tail winds, decreasing pressure, warm weather, and decreasing precipitation and cloud cover in spring [14], [17]. If migrant density patterns are determined by individual songbirds choosing to migrate on nights when they would expend less energy, it would follow that, for example, the energy costs of flying in calm air are lower than those of flying in strong winds or storms. However, due to the difficulty of studying free-flying avian physiology it has not yet been shown if or to what extent atmospheric conditions affect the energy costs of migrating birds. In other words, we do not know what the energetic consequences of flying on a low-migrant density night are. For this reason, we cannot predict how climate change and associated atmospheric changes will affect migrants' in-flight energy budgets and therefore variables such as stopover frequency and arrival date on the breeding or wintering grounds [14], [18].

**Figure 1:**
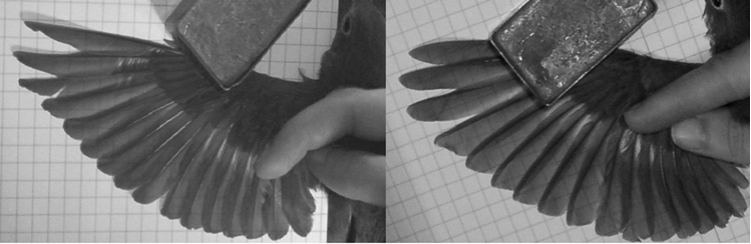
Wingtip shape in Swainson's Thrushes. To the left is a relatively pointed wing (C_2_ = 0.139) and to the right is a relatively round wing (C_2_ = 0.493). Grid squares in both pictures are 6.35 mm on each side.

This study was designed to determine which morphological and atmospheric variables, if any, affect the energy expenditure of small passerine migrants during flight in the wild. We used small (0.6–1.0 g) radio transmitters to measure the heart rates of 15 Swainson's Thrushes as they made migratory flights over the central United States (Illinois, Indiana and Wisconsin) in spring. Swainson's Thrushes are small (25–35 g), insectivorous passerines often used to study migratory behavior and physiology [19]. We used individual heart rate during the flights as a proxy for individual rate of energy expenditure; for birds, the relationship between heart rate and rate of energy expenditure is linear for a given activity and species: higher heart rates indicate higher rates of energy expenditure [10], [13], [20]. We measured thrushes prior to release (Figure 1), followed them with a radio-tracking vehicle, and recorded the signals from the transmitters as they migrated. Later, we obtained data on the atmospheric conditions during the flights using National Climactic Data Center data from the North American Regional Reanalysis model [21]. One drawback of using radio-transmitters is that we could not obtain exact altitudes for the birds and had to estimate them (see methods); thus, we could only approximate atmospheric conditions during the flights. We divided flights into initial ascent and cruise phases that we analyzed separately; data for a final descent phase were insufficient for analysis and we were unable to obtain heart rate during the initial ascent phase for one of the 15 thrushes. Finally, we used general linear models (GLM) to determine which morphological and atmospheric variables predicted average heart rate during each phase.

## Results

**Figure 2:**
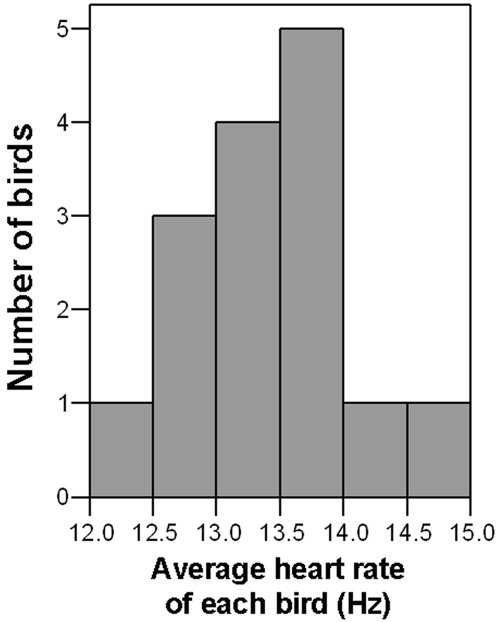
Frequency histogram for average heart rate. The graph shows the average heart rates (mean±s.e.m., 13.35±0.18 Hz) of 15 free-flying Swainson's Thrushes migrating over the central United States in spring after initial ascent and prior to final descent. Note the large amount of variation. The histogram for the initial ascent phase (not shown) is virtually identical except the mean is higher: 14.43±0.16 Hz.

Average heart rate varied considerably between individuals during both flight phases (Figure 2), and within flights was significantly higher during initial ascent than during cruise (paired *t*-test, N = 14, *t*_13_ = 9.71, *P*<0.001). During the initial ascent phase, thrushes with more pointed wingtips (*P* = 0.009) and lower wingloading (*P* = 0.008) had lower heart rates, consistent with predictions. The overall model was highly significant (N = 14, R^2^ = 0.650, *P* = 0.003), and the resulting equation was: heart rate (Hz) = 9.425+3.325*wingtip shape (C_2_, higher values indicate more rounded wingtips [3])+0.002*wingloading (gm^−2^). None of the atmospheric variables were significantly associated with heart rate during initial ascent.

**Figure 3:**
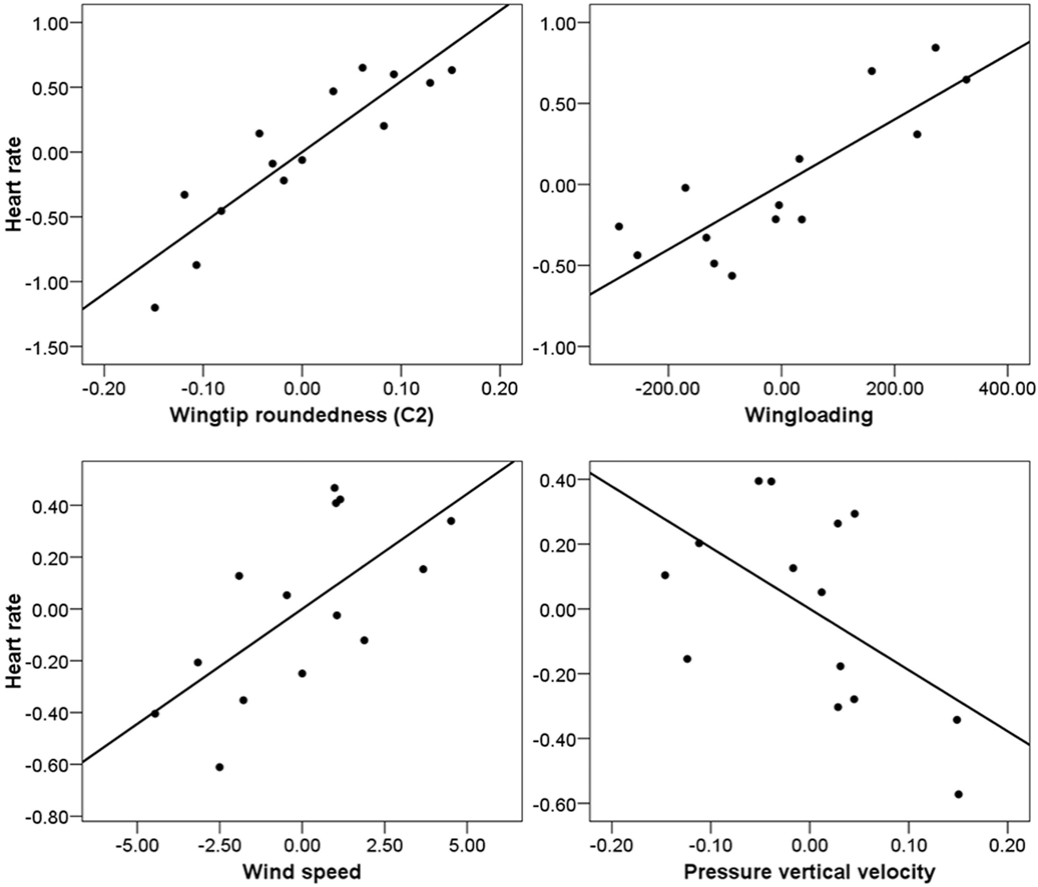
Partial regression plots for the cruise phase general linear model. The dependent variable in the analysis was average heart rate (Hz) during the cruise phase of 14 migratory flights. Heart rate (and thus energy expenditure) increased with increasing wingtip roundedness (C_2_), wingloading (gm^−2^), and wind speed aloft (ms^−1^), independent of wind direction. It decreased with increasing pressure vertical velocity (increasing atmospheric stability, Pas^−1^), although not significantly. Axes are unstandardized residuals.

During the cruise phase, wingtip pointedness (*P*<0.001), wingloading (*P* = 0.001), and average wind speed during the flights (*P* = 0.025) were significantly associated with heart rate; we also found a trend for pressure vertical velocity (*P* = 0.075), a measurement of large-scale atmospheric stability, to predict heart rate (Figure 3). Again, the overall model was highly significant (N = 14, R^2^ = 0.874, *P*<0.001). The equation was: heart rate (Hz) = 6.260+5.462*wingtip shape (C_2_)+0.002*wingloading (gm^−2^)+0.089*wind speed (ms^−1^)−1.890*pressure vertical velocity (Pas^−1^). Thus, heart rate increased with increasing wingtip roundedness, wingloading, wind speed, and decreasing pressure vertical velocity (decreasing large-scale atmospheric stability). We excluded one individual from this model for which we had no information on heading and for which we could therefore not obtain atmospheric variables; the parameter estimates were similar with and without this bird. Tarso-metatarsus length, temperature, precipitation and wind direction were not significant predictors of heart rate in either of the two models. However, the power of these last statistical tests was low: if one of the listed variables affected heart rate during flight, the probability of obtaining a significant result was 0.63 if the variable explained an additional 5% of the variation in heart rate. If the variable only explained an additional 1% of the variation, the probability of detecting a significant result was 0.11 [22].

## Discussion

Our data provide the first direct test of two predictions regarding the relationships between morphology and energy expenditure in naturally migrating, free-flying songbirds: Swainson's Thrushes with (*i*) more pointed wingtips and (*ii*) lower wingloading had lower heart rates and thus lower energy expenditure [20] than those with more rounded wingtips or higher wingloading. Furthermore, birds flying in high winds—regardless of wind direction—and on nights with negative pressure vertical velocity had higher heart rates during the cruise phase of the flight, although the latter result was not statistically significant. Negative pressure vertical velocity means that storms are developing and/or present, whereas pressure vertical velocity is positive when conditions are clear and calm [23].

Unfortunately, because we had to estimate the thrushes' altitudes, we could not obtain exact airspeeds for them and could therefore not address energy efficiency (the amount of energy used per distance flown relative to the surrounding air) in this study. However, the correlations we found between rate of energy use and morphological and atmospheric variables are interesting even if the mechanism by which they occur is that birds with certain morphology or birds flying in certain atmospheric conditions increase or decrease their airspeed. Our results are also correlative, but manipulating atmospheric parameters during natural migratory flight is impossible. Furthermore, experimental manipulations of wingtip shape would affect additional parameters, such as feather stiffness, shape and emargination, which also affect energy expenditure, so interpretation of experimental changes of wingtip shape may prove difficult. Thus, our results constitute one of the best possible tests of the influence of wing morphology and atmospheric dynamics on avian flight energetics in the wild.

It has long been accepted that the relatively pointed wingtips of migratory insects, bats and birds represent the outcome of selection for energy efficiency during migratory flight [3], [5]. Our study confirms that energy use varies with wingtip shape in free-flying songbirds. If this variation in energy expenditure translates into variation in fitness and if wingtip shape is heritable, which is likely because morphological variables tend to be highly heritable in songbirds [24], natural selection could cause the evolution of relatively pointed wings in migrants. Selection may act directly on energy use during migration, for example when individuals with depleted energy stores die while crossing large ecological barriers. However, selection may also act indirectly: individual birds with energy-saving morphological features should be able to fly further on a given amount of fuel and might therefore be able to increase their rate of migration [7], [25]. Migration is thought to be the most dangerous event in the annual cycle of small songbirds [26], so this would mean that individuals with favorable morphological characteristics would be exposed to relatively high mortality rates for a shorter period of time. Also, these individuals may arrive at their destinations earlier or in better condition than conspecifics, which could confer fitness benefits in terms of larger clutches, better territories, mates, and earlier breeding [7], [25], [27].

**Figure 4:**
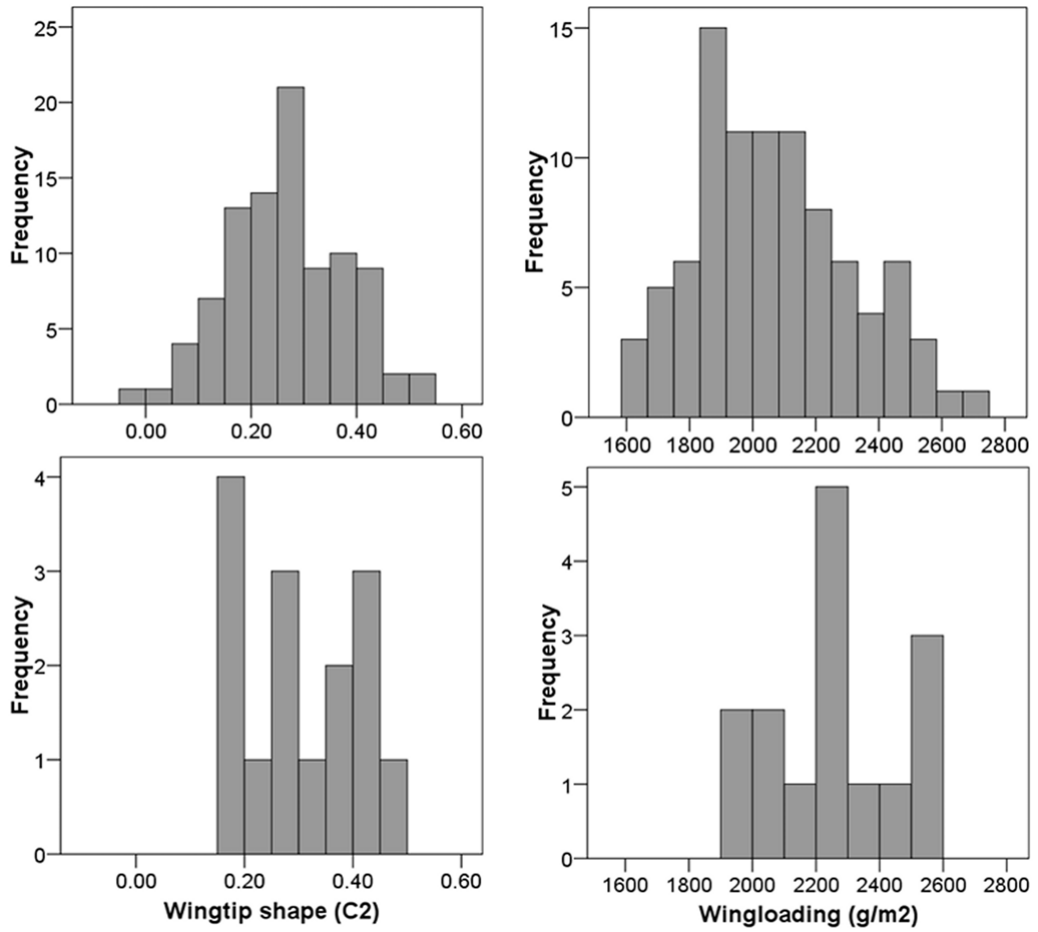
Frequency histograms for wingtip shape (C_2_, lower values indicate more pointed wings [3]) and wingloading. The top graphs show the wingtip shapes and wingloadings of 93 Swainson's Thrushes captured in Champaign-Urbana, IL in May 2003–2005 [25] while the bottom graphs show the same data for the 15 Swainson's Thrushes followed in this study. Average wingloading is slightly higher in this study than in the larger sample primarily because of the added mass of the transmitter.

Bowlin [25] found high variation in both wingtip pointedness (C_2_, −0.03 to 0.54) and wingloading (1585–2688 gm^−2^) in 93 Swainson's Thrushes stopping over in Illinois. The variation in wingtip shape and wingloading of our 15 thrushes was similar to the variation in this larger sample (Figure 4), although our birds' wingloadings were slightly higher due to the added mass of the transmitters and the fact that we avoided putting transmitters on thrushes in poor condition. Our cruise phase GLM analysis predicts a 3.2 Hz range of heart rates due to variation in wingtip shape for the larger sample; differences based on variation in wingloading would span a 2.2 Hz range. Preliminary data on the relationship between heart rate and energy expenditure during flight in Swainson's Thrushes (Bowlin et al. *unpubl. data*) suggests that this variation in heart rate would represent approximately a 22.7% and a 15.6% variation in predicted energy expenditure, respectively. The morphological variation Bowlin [25] observed is likely maintained by compromises between the lower energy requirements afforded by pointed wings and low wingloading and opposing selective pressures. Opposing pressures may include selection for take-off performance and maneuverability during foraging and escape flight [28]–[29], which favors more rounded wingtips, as well as the need to carry sufficient fuel loads in order to migrate successfully [8]. Also, an individual bird can adjust its wingloading prior to flight by gaining or losing mass [13], so it may not have consistently low or high wingloading throughout its migration.

**Figure 5:**
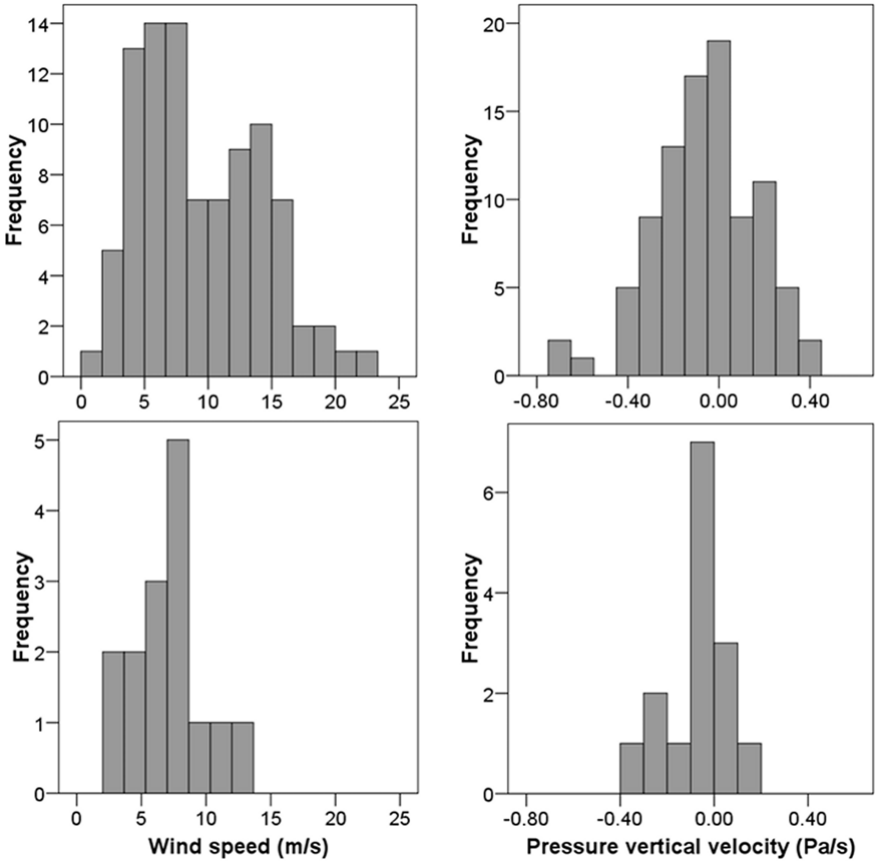
Frequency histograms for wind speed and pressure vertical velocity. The top graphs show the atmospheric conditions from the North American Regional Reanalysis Model [21] for 1–31 May, 2003–2005, at the 925 mb level at 0300 UTC (2100 local time) over the site where our thrushes were captured and released. The bottom graphs show the atmospheric conditions encountered by the 15 Swainson's thrushes in this study.

Our results indicate that atmospheric conditions, particularly wind speed, play an important role in the energetics of long-distance migration. The variation in atmospheric conditions the birds in this study flew in was similar to the overall variation in the atmospheric conditions available to the birds in May at a typical flight altitude (Figure 5), except that our thrushes did not fly on nights where there were high winds (>15 m/s). In accordance with this observation, and contrary to the predictions of aerodynamic theory [15]–[16], we found a positive correlation between wind speed, independent of wind direction, and energy expenditure. This suggests that thrushes use more energy in both high headwinds and high tailwinds. However, changes in aerodynamic power associated with changes in airspeed due to the presence of head- and tailwinds may be relatively small [30] and therefore undetectable given the low power associated with our statistical analysis. Error in estimating the birds' altitudes along with changes in wind direction and speed with altitude may have also prevented us from finding a relationship between wind direction and energy expenditure. Regardless, we do not yet understand the mechanism behind our primary observation. *Catharus* thrushes appear to maintain constant headings and airspeeds regardless of wind speed or direction [31], so it is unlikely that the observed increase in heart rate with increasing wind speed was due to individuals correcting for displacement by winds. Perhaps thrushes were responding to the fact that higher wind speeds, especially when associated with rapid vertical changes in wind speed or direction, are indicative of greater frequencies of both large- (on the order of kilometers to meters) and small-scale (on the order of meters to centimeters) turbulent eddies [32]. High winds or small-scale turbulence causing birds to work harder to maintain a constant heading could explain our results. Regardless, our findings are consistent with observations that Swainson's Thrushes avoid taking off when surface winds are high, whether wind direction is favorable or not [2].

In addition to wind speed, we found a trend for thrushes to use more energy when pressure vertical velocity was negative. A negative pressure vertical velocity measurement means that there is large-scale instability in the atmosphere: i.e. clouds are forming and/or rain is falling [23], although precipitation itself was not correlated with energy expenditure in our study. Clouds and rain form due to turbulent eddies in the atmosphere; therefore, as with high winds, it is possible that the mechanism for the observed result is an increase in small-scale turbulence. This could affect a bird's ability to fly steadily or maintain a constant heading and thus increase its energy expenditure. If lower atmospheric turbulence is the reason why thrushes use less energy in both lighter winds and clearer weather, songbirds might benefit energetically from migrating during the night. Turbulence is generally lower at night than during the day and laminar winds occur more frequently [32]–[33]. I.C.T. Nisbet proposed many years ago that many birds might migrate at night to avoid turbulence [34], and this idea has become more popular recently [35]. Our data appear to be consistent with the idea that birds do not only migrate at night to decrease water stress and/or avoid predation [36]–[37].

We speculate that climate change could affect the energy budgets of small intercontinental migrants if it involves adjustments in the frequency or severity of wind speed or atmospheric instability. Altering the energy budgets of migrants may affect the location of and need for stopover sites as well as change arrival times and advance or delay the onset of breeding in some species [7], [18], [25]. Particularly worrisome would be atmospheric changes over large ecological barriers such as the Sahara Desert, the Mediterranean Sea, the Gulf of Mexico, or the Pacific Ocean, as small birds often do not have much energy to spare when crossing these barriers [38].

## Materials and Methods

### Capture and measurement of birds

We mist-netted Swainson's Thrushes in a small (200×400 m) woodlot south of Urbana, Illinois (40°N, 88°W) in May 2003–2005. Once we captured a thrush, we measured the length of each primary feather on its left wing and its tarso-metatarsus length to the nearest 0.1 mm with dial calipers, its unflattened folded wing length to the nearest 1 mm with a wing rule, and its mass to the nearest 0.1 g with a spring scale. We also took wing photographs and used them to obtain wing area [25]. We used the lengths of primaries 2–9 to compute the size-constrained wingtip shape component C_2_ [3], which describes wingtip pointedness (Figure 1). Lower values of C_2_ indicate more pointed wingtips.

### Transmitter attachment and radiotracking

Our method of attaching heart rate transmitters (Sparrow Systems, Dewey, IL, designed by William W. Cochran) to captured thrushes is described in detail elsewhere [2]. In brief, each thrush was anesthetized and electrodes from the transmitter were placed under its skin through two small incisions before the transmitter was glued onto its back with eyelash adhesive. The methods used in this study were approved by Princeton University's IACUC and adhere to NIH standards and standards of the Animal Behavior Society for the use of animals in research.

Once we attached a transmitter, we allowed the thrush to recover from the surgery in a small, dark cage where we fed it mealworms and crickets *ad libitum*. We then released it back into the woods where it was captured and monitored it until it took off. We later used release mass to calculate its wingloading, even if it did not fly that night: thrushes were not recaptured once released. Once the thrush took off, we followed it with a radio tracking vehicle outfitted with a six-element Yagi antenna connected to a Falcon V (designed by William Cochran) or an AR 3000 (AOR Ltd., Tokyo, Japan) receiver in AM mode. The receiver was in turn connected to a Sony TD7 (Sony Inc., Tokyo, Japan) digital tape recorder. In addition to following the bird and recording the signal, we determined the bird's heading at least once per flight [31], and took notes on location and approximate altitude (based on whether or not various objects blocked the signal).

We followed nine of the 15 thrushes until they landed. The heart rates of the six thrushes lost during radiotracking did not significantly differ (*P*>0.05) from those of the other thrushes. The length of complete flights ranged from 1.0–6.4 h and averaged 3.1 h. We followed two of the six lost birds for >2 h and five for >1 h. Our results were qualitatively similar if we only included complete flights in the analyses.

### Signal analysis

We obtained heart rate from the transmitter signals using methods described elsewhere [19]. In brief, we used programs written by W. Cochran (Champaign, IL) which characterized the frequency modulation from the signal and then auto-correlated the modulation to obtain the period of the heart beats. The signal also provided wingbeat frequency; typically the program picked out heart rate (the higher frequency) more accurately, but variation in the signal required several (N = 3) records to be analyzed by hand because the program picked out wingbeat frequency more accurately than heart rate. Flights were divided into initial ascent and cruise phases based on changes in the bird's groundspeed and heart rate; the two phases were analyzed separately in case some variables only affected energetic expenditure during certain portions of the flight.

### Atmospheric variables

We obtained atmospheric data from the National Climactic Data Center's North American Regional Reanalysis model, freely available online [21]. We estimated the position of the bird during the flights based on notes taken while tracking. We used data for the 0300 UTC time step of the NARR (2100 local time) from the earliest takeoff (0246 UTC) until 0430, data for the 0600 time step from 0431–0730, data for the 0900 time step from 0731–1030, and data for the 1200 time step until the last bird landed at 1040 UTC (0440 local time). For the initial ascent phase, we used values for the initial time period (typically 0300 UTC) and location (40°N, 88°W) at the lowest pressure surface level (1000 mb). We chose pressure surface levels (i.e. altitudes) for each individual bird for the cruise phase by determining which level in the 1000–825 mb range best predicted bird heading from wind speed and direction and bird groundspeed and direction (Table 1). The average geopotential height of the pressure surface levels we chose for the 15 thrushes was 737 m. Once we chose an altitude, we obtained data from that pressure surface level for the various locations and time steps; we then averaged these data to obtain average weather conditions for the flights.

**Table 1:** Determining approximate flight altitude.

Pressure surface level (mb)	Predicted heading (°)
1000	77.9
975	59.9
950	25.0
925	9.5
900	24.5
875	8.7
850	15.2
825	36.5

Here we show how we determined the flight altitude of one of our Swainson's Thrushes. We used the bird's groundspeed and track direction as well as data on the winds aloft at the various pressure surface levels to predict the bird's heading, which could then be compared to its actual heading of 60°. It is clear from the table that the bird was flying near the 975 mb pressure surface level, so we used data from the 975 mb pressure surface level in our analysis of the effects of atmospheric conditions on heart rate. We performed this analysis for each of the 15 Swainson's Thrushes in this study to determine their flight altitudes.

The weather variables we used in the statistical models to predict heart rate were temperature (°C), precipitation (mm), pressure vertical velocity (Pas^−1^), wind speed (ms^−1^), and wind direction. We used two methods to examine wind direction: first, we measured the angle between the bird heading and the wind vector; next, we broke the wind vector into x and y components, where x winds were crosswinds and y winds were head/tail winds. Precipitation was not normally distributed, so we made it into a binary variable (0 = no precipitation, 1 = some precipitation during the flight). All other variables were distributed normally (Shapiro-Wilk tests, all *p*>0.05), and none of the predictor variables were significantly correlated with one another.

To estimate the larger set of atmospheric conditions available to the birds (Figure 5), we recorded the wind speeds and pressure vertical velocities from the North American Regional Reanalysis Model over the release site (88°W, 40°N) for 1–31 May 2003–2005. We chose the 925 mb level because it was closest to the average cruising altitude of the birds and 0300 UTC (2100 local time) since most of the birds took off within an hour of 2100.

### Statistical analyses

We analyzed the data using SPSS for Windows (Version 12.0.1). We constructed two separate general linear models (GLMs), one predicting heart rate during the initial ascent phase and the other predicting heart rate during the cruise phase, to determine which morphological and atmospheric variables were associated with heart rate during flight. We then used backward variable elimination to obtain the final models presented in the results and discussion section. Tarsus length, C_2_, wingloading, temperature, precipitation, pressure vertical velocity and wind speed and direction were the starting variables. The angle between wind direction and bird heading was not significant in either model, so we removed it and replaced it with y-wind and x-wind. These effects did not explain any additional variation when wind speed was in the model, indicating that the direction of the wind was unimportant; indeed, we removed wind speed from the cruise phase model to test this hypothesis and neither y-wind nor x-wind were significant predictors of heart rate.
